# Superior cluneal nerves radiofrequency in the management of chronic low back pain

**DOI:** 10.1016/j.inpm.2024.100428

**Published:** 2024-07-23

**Authors:** Leonado Arce Gálvez, Jesús Daes Mora, Rafael Enrico Valencia Gómez, José Luis Cuervo Pulgarín, David Hernández Abuchaibe, Christian Vladimir Guauque Marcelo

**Affiliations:** a Physical Medicine and Rehabilitation, Colombia; b Anesthesiology and Reanimation, Colombia; c Pain Medicine and Palliative Care, Fundación Universitaria de Ciencias de la Salud (FUCS), Bogotá, Colombia

**Keywords:** Radiofrequency, Pain management, Low back pain, Chronic pain

## Abstract

**Introduction:**

Chronic low back pain is a highly prevalent condition with multiple etiologies. Cluneal nerve neuropathy is an increasingly relevant condition in the management of this condition, and radiofrequency is an alternative management option.

**Methods:**

A case series, which included four patients who underwent ultrasound-guided conventional radiofrequency intervention of the superior cluneal nerves, using a previously undescribed technique and direction of intervention.

**Results:**

Patients reported a 50–90 % improvement in pain and a functional benefit for their daily activities of more than 40 % at 4- and 10-week follow-up, with no adverse events following the intervention.

**Conclusions:**

Continuous radiofrequency of the cluneal nerves is an interesting alternative in the management of this pathology of low back pain. The ultrasound technique described may be a management proposal with lower risk and adequate effectiveness.

## 1 Introduction

Chronic low back pain is a condition of high prevalence worldwide, it has different etiological sources ranging from musculoskeletal to neurological causes; however, the varied sources of pain are a challenge when approaching patients suffering from this type of pain [1]. The therapeutic approach to chronic low back pain is multidisciplinary and can include different types of pharmacological and non-pharmacological interventions [2].

Among all causes of pain consultation, patients are often found with sensory changes in the lumbosacral region, but they do not follow a radicular pathway, which is why the territories of innervation of the peripheral nerves are increasingly considered. Peripheral nerves can become trapped in their path through fascial layers, including the fibro-osseous tunnel over the iliac crest [1,2].

A condition which has become more prominent in recent years is cluneal nerve entrapment neuropathy, in this entity, the sensory innervation of the lower back at the level of the iliac crest and the gluteal region is affected. The cluneal innervation is divided into superior, middle, and inferior nerves. Entrapment of the superior cluneal nerves is the most common condition and can manifest as a symptom of pain in the lower back and gluteal region with or without pain to the extremity [3]. The diagnosis of this condition is purely clinical, evaluating the sensory distribution of pain and the evocation of neuropathic pain on palpation of the nerve emergence over the iliac crest [4]. Interventions when cluneal nerve neuropathy is suspected are considered to range from conservative management with rehabilitation and neuromodulatory drugs to analgesic interventionism [3].

Interventional image-guided blocks, either fluoroscopy or ultrasound, have proven to be clinically useful in relieving these symptoms [5]. However, when local anesthetics and anti-inflammatory drugs are applied, the analgesic effects tend to be limited in time; at this point an interesting intervention tool is conventional radiofrequency, where in the case of the superior cluneal nerves, having a sensory component without motor innervation, it can be neurolytic, thus seeking a prolonged analgesic effect.

## 2 Case series

A case series including four adult patients from Bogotá, Colombia, was carried out. They were diagnosed with superior cluneal nerve entrapment neuropathy as the origin of their low back pain. Patients had been diagnosed at least six months prior to the first intervention and had symptoms evoked by palpation of the nerve emergence at the iliac crest (7 cm laterally from the spinous process of L4). To perform the diagnosis of neuropathic pain, the diagnostic tool of the neuropathic pain interest group of the International Association for the Study of Pain was used. These criteria include four items, at least three of which must be present for a diagnosis to be made. They include a cause of central or peripheral neurological tissue injury, a plausible neuroanatomical distribution, sensory symptoms and an imaging or electrophysiological study confirming the injury.

They had received pharmacological management with gabapentinoids and 1-week cycles of NSAIDs at therapeutic doses in addition to having attended at least 20 sessions of physiotherapy based on stretching, muscle strengthening, massage, and ultrasound, without significant clinical response. A diagnostic block was performed 6 weeks prior to radiofrequency, which resulted in an improvement of more than 50 % of the symptoms for at least one week. For this procedure, ultrasound guidance was used with a 5–13 Mhz linear transducer, performing a single puncture at the emergence of the superior cluneal nerves in the iliac crest at 7 cm lateral to the spinous apophysis of L4, applying a total volume of 2 cm of 0.25 % bupivacaine plus 2 mg of betamethasone. None of the patients had hematologic or oncologic conditions that contraindicated the intervention. all patients signed an informed consent for the performance of the procedures and the use of their clinical information.

Two scales were used to follow up with the patients. In the numerical rating scale, the patient described the intensity of his pain from 1 to 10, with 10 being the most intense pain [6]. Considering that these are subjective values, additional measurement was performed with the functional rating index that asks questions about the characteristic of the patient's pain and qualifies the degree of limitation from 0 to 100 %, with a higher degree of limitation due to pain values close to 100 [7].

**Fig. 1:**
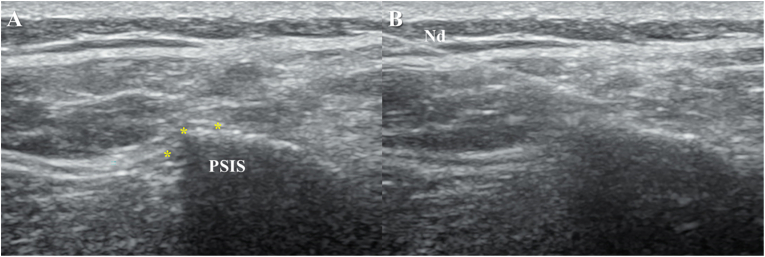
A-anatomical location and purpose of superior cluneal nerves. B- Intervention and needle position. PSIS: posterior superior iliac spine. *: Radio frequency needle target. Nd: Needle.

The conventional radiofrequency technique was performed with an ultrasound machine with a 5–13 Mhz linear transducer, because previous descriptions of this procedure are performed with fluoroscopic guidance, the approach proposed by our pain medicine department was used. The technique consists in identifying the emergence of the cluneal nerves in the iliac crest, by positioning the transducer 7 cm laterally to the right or left of the spinous process of L4, at three different points around this location, in a plane projection (Fig. 1), in this plane, the convex bony surface and the thoracolumbar fascia are appreciated, where three lesions are sought to cover this convexity, one below the insertion of the fascia in the bone and two above covering the entire path of the superior cluneal nerves.

We proceeded to advance a 22G radiofrequency needle with an active tip of 10 mm in plane until reaching the target of intervention. Afterwards, 1.5 cubic centimeters of 3 % saline solution was applied to increase the size of the radiofrequency lesion, then 0.5 cubic centimeters of 2 % lidocaine was administered and a conventional radiofrequency lesion with a time of 120 seconds at 80 °C was performed, repeating these same parameters for the 3 lesion points (image 1). The adequate positioning of the needle was verified at each point with ultrasonography, the iliac crest was considered as a reference where most of the superior cluneal nerve entrapments occur. A sensory stimulation test was additionally performed before each lesion, obtaining a response between 0.3 and 0.5 V in all patients. After each intervention, the patients were observed for 30 minutes and were discharged. An assessment of pain intensity and medication with a functional scale for low back pain was performed 30 minutes before the intervention and control at four and ten weeks (Table 1).

**Table 1:** Patients included in case series and therapeutic outcomes.

Side left/right	NLS left///right	Age	Sex	Initial NRS	NRS post procedure	NRS at 4 weeks	NRS at 10 weeks	Initial FRI.	FRI at 4 weeks	FRI at 10 weeks	Temp°C/T(s)/Dur(ms)/Freq(Hz)/Volt(v)
Right	0///3	60	Male	8/10	4/10	2/10	1/10	57,5	37,5	25	80 °C/120s/20 ms/2Hz/45v
Bilateral	3///3	40	Female	8/10	2/10	4/10	4/10	67,5	45	40	80 °C/120s/20 ms/2Hz/45v
Bilateral	3///3	64	Male	8/10	2/10	0/10	0/10	80	5	0	80 °C/120s/20 ms/2Hz/45v
Bilateral	3///3	51	Female	9/10	1/10	2/10	0/10	65	27,5	5	80 °C/120s/20 ms/2Hz/45v

**NLS:** Number of lesions per side. **NRS:** Numerical rating scale. **FRI:** Functional rating index. **Temp°C:** Temperature in Celsius. **T:** Time in seconds. **Dur:** Duration in milliseconds. **Freq:** Frequency in Herz. **V:** Voltage in volts.

**Fig. 2:**
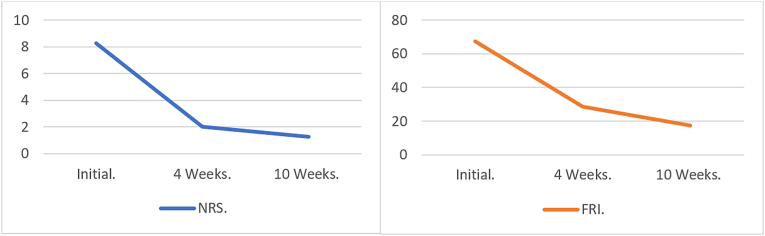
Numerical rating scale (NRS) and functional rating index (FRI) measurements and monitoring over time.

All the patients showed a symptomatic improvement between 50 and 90 % of the symptoms in the four-week control. In addition, an improvement in the functional scale of 40 % was found; these results were similar in the ten-week control. A decrease in the pain scale and an improvement in functional performance were reported, with a great impact on their activities of daily living, including sports and work. (Fig. 2). No patient presented secondary events or increased pain and continued their treatment with a home rehabilitation plan focused on stretching and muscle strengthening.

## 3 Discussion

Superior cluneal nerve entrapment neuropathy is an increasingly relevant condition in the differential diagnosis of chronic low back pain. In our case series, we illustrate the follow-up in pain and functional condition of four patients who underwent continuous radiofrequency of the superior cluneal nerves. It should be considered for this intervention that it is essential to perform a therapeutic diagnostic block to confirm the source of the pain, although the success of the diagnostic block does not predict radiofrequency releases, an accurate diagnosis favors positive releases [5]. In conventional radiofrequency, a neurolytic lesion is achieved based on the temperature reached by the active tip, which in the case of the cluneal nerves, which are purely sensory, interrupts nociceptive conduction and improves pain.

Conventional radiofrequency interventions in patients with superior cluneal nerve neuropathy have been reported in the past [8]. These studies have highlighted the safety of the intervention and the clinical effectiveness obtained in patients. However, the previously reported studies do not include functional measurements of the patients and have used only fluoroscopic guidance [[8], [9], [10]]. Therefore, considering this procedure with ultrasonographic imaging guidance is appropriate and provides less radiation exposure for the patient and the medical staff. The definition of an ultrasonographic reference based on nerve entrapment in its passage through the iliac crest and thoracolumbar fascia may favor a positive outcome since it is a critical anatomical point and is a frequent point to detect symptoms on physical examination.

Our case series is the first publication that proposes an effective technique for the treatment of superior cluneal nerve neuropathy with conventional radiofrequency with ultrasound guidance. The anatomical references and ultrasound targets used in this series have been previously used in fluoroscopy or diagnostic blocks, but without being taken into account in the performance of radiofrequency or considering the nerve coverage at three points on the bone surface described in our service [5,10]. Although there is a technique for direct visualization of the nerves in relation to the lumbar square muscle, the procedure was performed with reference to the iliac crest and the thoracolumbar fascia, because this is the point of perforation of the nerves where the traps are generated. In addition, a sensitive response was obtained in the stimulation that confirms this reference as an interesting point to consider. Given the efficacy found, it is considered that the references are the indicated ones in this type of procedures without radiation risk for the physician and the patient, with the possibility of dynamic observations in real time. Our work is limited by its small number of patients, but its efficacy and the absence of adverse events stand out.

## 4 Conclusion

Conventional radiofrequency for the treatment of the superior cluneal nerves as a cause of low back pain is an effective therapeutic tool that should be considered as a differential diagnosis. This case series offers a new ultrasound technique to address this pathology.

## Funding

The authors did not receive funding to carry out this study.

## Declaration of competing interest

The authors declare that they have no known competing financial interests or personal relationships that could have appeared to influence the work reported in this paper.
